# The Mediator Effect of Personality on the Relationship Between Symptomatic Impairment and Treatment Outcome in Eating Disorders

**DOI:** 10.3389/fpsyg.2021.688924

**Published:** 2021-07-02

**Authors:** Laura Muzi, Laura Tieghi, Anna Franco, Michele Rugo, Vittorio Lingiardi

**Affiliations:** ^1^Department of Dynamic and Clinical Psychology, and Health Studies, Sapienza University of Rome, Rome, Italy; ^2^Eating Disorder Clinic “Residenza Gruber,” Bologna, Italy

**Keywords:** personality, eating disorders, symptom severity, comorbidity, therapy outcome

## Abstract

Features of personality disorders (PDs) have been found to explain meaningful variance in the onset, maintenance, and symptomatic presentation of eating disorders (EDs), and a co-occurent personality pathology is commonly associated with poorer response to ED treatment. The “pathoplasty model” of the relationship between personality and EDs implies that, once both conditions are established, they are likely to interact in ways that modify therapy outcome; however, to date, no studies have explored overall personality functioning, and especially PD clusters, as a mediator of treatment outcome. The present study aimed at conjointly exploring the associations between personality functioning and PDs, respectively, with pre-treatment ED symptomatic impairment and therapy outcome; and the mediating role of personality variables. At treatment onset, a sample of 107 women with ED problems were evaluated using both the Structured Clinical Interview for DSM-5 (SCID-5-CV) and the Shedler-Westen Assessment Procedure-200 (SWAP-200)—a clinician-rated procedure to dimensionally assess personality. Participants were also asked to complete self-report questionnaires on overall ED symptomatology, symptoms of binge eating and purging behaviors, and therapy outcome. The findings showed that, over and above the categorical ED diagnosis, the SWAP-200 healthy personality functioning score mediated the relationship between baseline ED symptom severity and therapy outcome, as well as the association between baseline bulimic symptoms and treatment outcome; furthermore, SWAP-200 Cluster B PD scores mediated the link between baseline binge eating and purging symptoms and therapy outcome, whereas scores in Clusters A and C showed no significant effects. The findings suggest that personality-based outcome research may improve treatment effectiveness in this difficult-to-treat population.

## Introduction

Eating disorders (EDs), including anorexia nervosa (AN), and bulimia nervosa (BN), are commonly ranked amongst the most complex and pernicious of all psychiatric illnesses to treat. Often, they have a chronic course, and in some cases, they are fatal. A significant therapeutic challenge is that, in patients with a severe ED, psychiatric comorbidities are the rule, rather than the exception (e.g., Keski-Rahkonen and Mustelin, 2016; Halmi, 2018). Several studies and systematic reviews have suggested that the lifetime prevalence of an additional psychiatric disorder is between 55–80% for AN and 85–95% for BN (Hudson et al., 2007; Van Alsten and Duncan, 2020). With estimated comorbidity rates of 27–93% (with higher rates registered by inpatients and those receiving intensive treatment), personality disorders (PDs) are among the most prevalent co-occurring conditions (Cassin and Von Ranson, 2005).

“Personality” describes a set of relatively stable ways of thinking, feeling, behaving, and relating to others, resulting from the convergence of constitutional factors, development, and social and cultural experiences (Lingiardi and McWilliams, 2017; McWilliams et al., 2018). This variable has been found to influence a wide range of psychiatric disorders, as well as patients’ motivation, compliance, and response to treatment (Ramos-Grille et al., 2013; Steinert et al., 2015; Bagby et al., 2016; Huber et al., 2017). The relationship between personality, personality traits or disorders, and EDs has received considerable empirical testing, with linkages found between personality and ED etiology, symptomatic expression, and maintenance (Farstad et al., 2016; Martinussen et al., 2017). Lilenfeld et al. (2006) outlined that personality and EDs may interact in a variety of ways, and proposed several conceptual models to describe potential causal or correlational relationships between them. Of note, the so-called *pathoplasty model* implies that, once personality traits or disorders and EDs are established, they may influence each other in ways that modify the presentation and course of each condition, including ED symptomatic impairment and treatment outcome. This model is in line with the perspective that patients’ personality is a relevant “context” (Westen et al., 2006) in which ED symptoms serve different functions and provide alternative meanings.

Although research on personality as a predictor of ED outcome is sparse, some relevant studies, drawing on the pathoplasty model, have found that PDs are commonly associated with poorer response to treatment (Steinhausen, 2002, 2009; Thompson-Brenner and Westen, 2005; Wildes et al., 2011; Muzi et al., 2020, 2021). Further research has suggested that high attrition, low compliance, dropout, symptom chronicity, low recovery rates, and low efficacy of therapeutic interventions relate to the personality characteristics of individual patients (Pham-Scottez et al., 2012; Martinez and Craighead, 2015; Levallius et al., 2016). ED patients with comorbid personality pathology have been found to present more severe overall psychopathology (according to anxiety, depression, somatization, psychoticism, and global severity scales) (e.g., Wonderlich et al., 1994) and higher levels of ED symptoms (Westen and Harnden-Fischer, 2001; Hopwood et al., 2010). More specifically, some outcome studies have found borderline personality disorder comorbidity and other Cluster B personality traits to predict negative therapy outcomes (Fahy et al., 1993; Rossiter et al., 1993; Voderholzer et al., 2021). Furthermore, follow-up studies with mixed ED samples have shown that baseline borderline symptoms relate to lower overall functioning, higher levels of ED symptoms, and lower rates of therapeutic change and life satisfaction (Sansone and Fine, 1992; Wonderlich et al., 1994). On the other hand, patients with a borderline personality disorder and a comorbid ED have been found to have a greater risk of recurrent suicide attempts, an increased risk of recurrent non-suicidal self-injury, and lower rates of remission (Zanarini et al., 2006; Chen et al., 2009). Interestingly, a follow-up study found that borderline personality disorder predicted a more negative ED outcome when measured dimensionally, but not when measured categorically (Wonderlich et al., 1994).

Research has also found that perfectionism is a core feature of severe EDs (Martinez and Craighead, 2015), and potentially predictive of the onset of eating pathology (Fairburn et al., 1999; Halmi et al., 2005). More general obsessive-compulsive personality traits have been shown to be a negative prognostic feature among ED patients (Steinhausen, 2002; Lilenfeld et al., 2006) that tend to persist after ED recovery (von Ranson et al., 1999; Sutandar-Pinnock et al., 2003). A systematic review also suggested that this variable may moderate or mediate the outcome of ED treatment, especially in patients with AN (Crane et al., 2007). Despite these findings, there is very scarce evidence with respect to other PDs, within Clusters A, B, or C. However, some authors have argued that the presence of suspiciousness, paranoid features, and interpersonal distrust or detachment may interacted with other patient variables to predict ED symptoms over the long term (Dingemans et al., 2016), and a recent study also found that paranoid and schizoid personality traits may predict therapy outcome in residential treatment for ED patients (Muzi et al., 2020). Moreover, avoidant-insecure personality features and a diagnosis of avoidant personality disorder have been found to be associated with more severe symptomatic impairment, lower ED symptom improvement, and higher treatment utilization (Thompson-Brenner et al., 2008a; Vrabel et al., 2010).

Some limitations of these contributions should be noted. First, none of the aforementioned studies with ED samples examined overall personality functioning—or PD cluster—as a mediator or moderator of treatment outcome (Linardon et al., 2017), despite some preliminary findings showing the mediating role of personality in the relationship between patients’ attachment styles and ED symptomatic presentation (Eggert et al., 2007; Münch et al., 2016). Furthermore, research into the role of personality traits or disorders and their link to EDs is necessarily complicated by the ongoing debate over whether PDs are best conceptualized categorically or dimensionally, with increasing support for the latter hypothesis (e.g., Widiger, 2007). In this vein, some authors have outlined that the investigation of personality within the categorical boundaries of ED diagnoses, as proposed by the *Diagnostic and Statistical Manual of Mental Disorders* (DSM-5; American Psychiatric Association, 2013), could be limited by the high rates of residual diagnoses (Fairweather-Schmidt and Wade, 2014), the common “diagnostic cross-over” between the main ED diagnoses, the low temporal stability of the main ED diagnoses, and their lack of discriminant validity in terms of severity of symptomatic impairment (Eddy et al., 2008). More specifically, with respect to treatment outcomes, some studies have found that categorical DSM-5 ED diagnoses do not predict patients’ responses to therapeutic interventions or future clinical courses (Westen and Harnden-Fischer, 2001; Raykos et al., 2018; Muzi et al., 2021), and that DSM-5 severity specifiers are not related to ED psychopathology, overall impairment, health status, comorbid conditions (e.g., depressive symptoms), or therapy outcomes (Gianini et al., 2017; Machado et al., 2017; Dalle Grave et al., 2018). Conversely, studies employing more dimensional and empirically derived approaches, such as the Shedler-Westen Assessment Procedure-200 (SWAP-200; Westen and Shedler, 1999a,b), have shown promising results in the identification of personality constellations in ED patients (Westen and Harnden-Fischer, 2001; Thompson-Brenner and Westen, 2005; Gazzillo et al., 2013), including healthy personality features and psychological resources (e.g., Muzi et al., 2020), as well as their predictive value in determining therapeutic outcomes (Thompson-Brenner et al., 2008a,b).

Drawing on the literature, the present study aimed at conjointly exploring the associations between personality functioning and PD features, respectively, with pre-treatment ED symptomatic impairment and therapy outcome; and the mediating role of personality features. More specifically, we tested the following hypotheses:

(a) There would be significant positive associations of moderate magnitude (Cohen, 1988) between PDs in Clusters A, B, and C, more severe pre-treatment ED symptomatic impairment, and worse therapy outcome (e.g., Farstad et al., 2016), as well as a significant association between healthy personality functioning (see the SWAP-200 description in the “Measures” section), lower severity of ED symptoms at treatment intake, and better therapy outcome;
(b) There would be no significant differences between the main DSM-5 ED diagnoses (AN and BN) in terms of baseline ED symptomatic impairment and therapy outcome, in line with studies supporting the overall lack of discriminant validity of ED categorical diagnoses (e.g., Westen and Harnden-Fischer, 2001; Raykos et al., 2018); and
(c) Overall healthy personality functioning and PD clusters would mediate the association between the severity of ED symptomatic impairment at baseline and therapy outcome, in line with previous theoretical contributions (e.g., Lilenfeld et al., 2006) and empirical evidence showing that personality may influence both the severity or pattern of symptomatology and the course of the illness (e.g., Thompson-Brenner and Westen, 2005; Muzi et al., 2020).

## Materials and Methods

### Participants

Participants were patients who had been consecutively admitted to a specialized, intensive, and psychodynamic-oriented residential treatment center for ED in Bologna (Italy) between September 2017 and April 2020. The inclusion criteria were: (a) at least 18 years of age; (b) a diagnosis of DSM-5 AN or BN, established at intake by the consensus of a licensed staff psychiatrist and a clinical psychologist, based on the Structured Clinical Interview for DSM-5 (SCID-5-CV; First et al., 2016); (c) cisgender women; and (d) no organic syndrome, psychotic disorder, or syndrome with psychotic symptoms that could complicate the assessment of any study variable.

An initial sample of *N* = 144 met these criteria. Twenty-one patients (14.6%) were excluded due to premature discharge or dropout and 16 patients (11.1%) were not considered due to missing data at intake or discharge. Out of the final study sample of *N* = 107 patients who completed all assessment measures at treatment intake and discharge, 62 (57.9%) were diagnosed with AN, with an average baseline BMI of 16.12 kg/m^2^; and 45 (42.1%) fulfilled the diagnostic criteria for BN, with an average BMI of 21.95 kg/m^2^. Participants’ mean age was 24.06 years (*SD* = 8.32), and all were White. Most patients were single or separated (*N* = 103, 96.2%) and had no previous instance of hospitalization in a psychiatry unit (*N* = 82, 76.2%). At treatment intake, 86 patients (80.4%) reported the presence of dietary restrictions in the previous week, 51 patients (47.6%) reported compensatory behaviors, and 44 patients (41.1%) reported the presence of binge episodes. Their mean age of ED onset was 16.08 (*SD* = 3.64). The majority of patients (*N* = 74, 69.1%) also showed at least one comorbid PD, as assessed by the SCID-5-CV. More specifically, 35 patients fulfilled the diagnostic criteria for borderline personality disorder (32.7%), 21 for obsessive-compulsive personality disorder (19.6%), 10 for avoidant personality disorder (9.3%), 3 for schizoid personality disorder (2.8%), 2 for paranoid personality disorder (1.9%), 1 for antisocial personality disorder (0.9%), 1 for narcissistic personality disorder (0.9%), and 1 for dependent personality disorder (0.9%). Additionally, 6 patients (5.6%) received an “other specified” or “unspecified” personality disorder diagnoses. Furthermore, 18 patients (16.8%) received a concurrent diagnosis of major depressive disorder, 16 (14.9%) an anxiety disorder, 14 (13.1%) obsessive-compulsive disorder, 6 (5.6%) a persistent depressive disorder (dysthymia), 3 (2.8%) an “other specified” or “unspecified” depressive disorder, and 2 (1.8%) a somatic symptoms disorder. Eight patients (7.5%) also reported an alcohol use disorder, and 5 (4.7%) reported another substance-related disorder.

Seven therapists (all cisgender women) participated in the study as raters of patients’ personality functioning and disorders. Their mean age was 42.7 (*SD* = 3.76; range = 37–49). The main self-reported clinical orientations were psychodynamic (*N* = 6, 85.7%) and cognitive behavioral (*N* = 1, 14.3%); all were clinical psychologists. The average length of clinical psychotherapy practice was 10.1 years (*SD* = 3.07; range = 7–15) and the average time spent per week practicing psychotherapy was 22.7 h (*SD* = 5.14; range = 15–35). All of the present study data were added to those of the patients and clinicians who participated in previous investigations using the same assessment tools (Muzi et al., 2020, 2021).

### Measures

Sociodemographic and baseline clinical characteristics (e.g., age, marital status, length of stay in residential treatment, age of ED onset, dietary restrictions, etc.) were available from each patient’s clinical chart.

#### Structured Clinical Interview for DSM-5, Clinical Version (SCID-5-CV)

The SCID-5-CV (First et al., 2016) is a semi-structured interview that was designed to categorically assess psychopathology according to the DSM-5. It is typically administered by a clinician who is familiar with the DSM-5 diagnostic criteria. Interview questions are provided alongside each DSM-5 criterion to aid users in rating each criterion as either present or absent. The previous version of the interview (SCID-IV) showed good interrater and test–retest reliability (Lobbestael et al., 2011).

#### Shedler–Westen Assessment Procedure-200 (SWAP-200)

The SWAP-200 (Westen and Shedler, 1999a,b; Shedler et al., 2014) is a well-established psychometric procedure that was designed to provide a comprehensive assessment of patient personality pathology and overall personality functioning. Unlike most personality assessment instruments, the SWAP-200 is designed for use by clinicians and mental health professionals in the context of a thorough examination of a patient in treatment. This Q-sort instrument consists of a set of 200 personality-descriptive statements, written in straightforward, jargon-free language, to be used by clinicians with varying theoretical orientations and levels of experience. Clinicians are asked to rank-order items for their degree of applicability to the patient at hand, and they must assign each rank or score a specified number of times (e.g., limiting the highest-ranking scale points [5, 6, or 7] defined as most descriptive of the patient to a smaller number of items, while assigning lower scores to a higher number of items). The SWAP-200 assessment provides: (a) a personality diagnosis that matches the patient assessment with 10 PD Scales that are prototypical descriptions of DSM–5 PDs and (b) a personality diagnosis based on the correlation/matching of the patient’s SWAP-200 description with 11 empirically derived Q-factors/styles of personality. A “healthy personality functioning” score, which reflects experts’ consensual understanding of adaptive personality functioning, is also provided (Westen and Shedler, 1999a). The measure yields both categorical and dimensional diagnoses. When a categorical diagnosis is desired, *T* > 60 indicate that a diagnosis applies and *T* > 55 indicate the presence of clinically significant “features.” If more than one scale has a *T*-score above 60 and the healthy personality functioning scale has a *T* < 60, the highest score provides the primary personality diagnosis. However, in line with the research aims and the growing consensus on the limitations of contemporary categorical conceptualizations of personality, the present study used only the dimensional scores of the PD scales and healthy personality functioning. The SWAP-200 has been shown to have very good validity and reliability, both with raters who have not been trained in using the instrument (Shedler and Westen, 2004; Blagov et al., 2012) and with those who have received instrumental training (Bradley et al., 2007). In the present study, Cronbach’s alphas for each SWAP-200 scale ranged from 0.72 to 0.85.

#### Eating Disorder Inventory-3 (EDI-3)

The EDI-3 (Garner, 2004; Giannini et al., 2008) is a self-report questionnaire that is widely used in both research and clinical settings to assess the core components of eating psychopathology. It consists of 91 items organized into 12 primary scales, consisting of 3 ED-specific scales and 9 general psychological scales that are highly relevant to EDs. It also yields six composite scores: one that is ED-specific and five that refer to general integrative psychological constructs. In this study, we considered the Global Psychological Maladjustment composite (GPMC) score as an index of overall ED symptomatic impairment. The EDI-3 was found to yield adequate convergent and discriminant validity (Clausen et al., 2011). In the present sample, Cronbach’s alphas for EDI-3 scores ranged from 0.70 to 0.94.

#### Bulimic Investigatory Test, Edinburgh (BITE)

The BITE (Henderson and Freeman, 1987; Orlandi et al., 2005) is a 33-item, binary yes/no response self-report questionnaire aimed at assessing and identifying bulimic symptoms and behaviors. The instrument has been previously used for the early detection of bulimic symptoms in the general population, to assess the intensity of the pathology, and to register responses to treatment. It consists of two subscales: the Symptom Scale (30 items) (used in the present study), which measures the degree of symptomatic impairment; and the Severity Scale (3 items), which provides an index of the frequency of binge eating and purging behaviors. In the present sample, Cronbach’s alpha for the BITE Symptom Scale was 0.79.

#### Outcome Questionnaire-45.2 (OQ-45.2)

The OQ-45.2 (Chiappelli et al., 2008; Lambert et al., 2010) is a 45-item self-report instrument that was designed to measure important areas of functioning (i.e., symptoms, interpersonal problems, social role) that are of central interest to mental health. Each item is rated on a 5-point Likert scale ranging from 0 (*never*) to 4 (*almost always*). The sum of item scores (after reverse coding selected items) provides a total score, which was used in the present study as the outcome index. In prior studies, the measure has been found to demonstrate good internal consistency and test–retest reliability (Doerfler et al., 2002). In the present study, Cronbach’s alpha for the OQ-45.2 total score was 0.90.

### Procedures

Patients were attending an intense, multimodal, residential treatment program with a main psychodynamic orientation, which included both group and individual psychotherapy. Average treatment length was 6.08 months (*SD* = 2.15, range = 3–13). According to the most widespread practice guidelines for ED treatment, a team approach and a patient-tailored perspective were the cornerstones of the therapeutic program (Yager et al., 2006). Thus, a multidisciplinary team of specialized professionals (i.e., psychiatrists, psychologists, social workers, nutritionists, physicians, nurses) was involved. Each patient received individual psychotherapy once or twice a week on the basis of a comprehensive examination of his or her psychological development, psychodynamic issues, cognitive style, comorbid psychopathology, and family situation.

During the first week of treatment, all patients were evaluated with the SCID-5-CV by a licensed staff psychiatrist and a clinical psychologist, to ensure fulfillment of the inclusion criteria. Additionally, height and weight were measured during a full medical examination, to calculate BMI. Moreover, at the same time point and during the last week before discharge, all patients who agreed to participate were asked to complete self-report measures to assess ED-specific symptoms and therapy outcome, evaluated in terms of overall psychopathological impairment. To minimize the effect of acute starvation and acute ED symptoms on personality, the SWAP-200 assessment was provided by treating clinicians within the first 2 weeks after admission. Psychotherapists were trained to use the SWAP-200 in a 16-h workshop led by the first and last authors of this article. In their baseline evaluations of personality functioning and disorders, treating clinicians were blind to patients’ SCID-5-CV assessments (administered by other staff members) and all other study variables, except for participants’ ED symptoms. All study subjects participated voluntarily and provided written informed consent prior to the assessments, following the review and approval of the study protocol by the local research ethics committee.

## Statistical Analyses

All analyses were performed using SPSS Version 25 for Windows and the *jAMM* package of the statistical software Jamovi, based on R (R Development Core Team, 2018). To test the first hypothesis, bivariate correlations (Pearson’s *r*, two-tailed) were calculated to study the relationship between PDs and healthy personality functioning (assessed by the SWAP-200), baseline overall ED symptomatic impairment (evaluated by the EDI-3 GPMC score), and bulimic symptoms (assessed by the BITE). Partial correlations were then carried out to explore the relationships between personality variables and therapy outcome, controlling for baseline values (using the OQ-45.2 total score). Due to the high rates of PD comorbidity, data were analyzed at the cluster level (Clusters A, B, and C). More specifically, for each patient, average scores of the SWAP-200 PD scales comprising each cluster were computed (i.e., Cluster A: paranoid, schizoid, and schizotypal PD scales; Cluster B: antisocial, borderline, histrionic, and narcissistic PD scales; Cluster C: avoidant, dependent, and obsessive-compulsive PD scales). To test the second hypothesis, group differences (in terms of the DSM-5 main diagnostic categories, AN and BN) on baseline ED symptomatic impairment and therapy outcome were analyzed using a multivariate analysis of covariance, controlling for age and BMI (MANCOVA). Finally, separate mediation models of the relationship between pre-treatment symptomatic impairment (EDI-3 and BITE overall scores) and therapy outcome (OQ-45.2) were tested to identify the mediation effects of overall personality functioning and PD clusters on these relationships. Because therapeutic change was the outcome variable of interest, a residualized change score was calculated for the OQ-45.2 total score by running a linear regression with the discharge values as the outcome and the baseline scores as the predictor. The standardized residual values were then saved and used in subsequent analyses. According to contemporary contributions on mediation analyses (Hayes, 2009; Hayes and Rockwood, 2017), the *indirect effect* of a predictor variable X on the outcome variable Y through a mediator M quantifies the estimated difference in Y resulting from a one-unit change in X through a sequence of causal steps in which X affects M, which in turn affects Y. In the present study, the bias-corrected 95% confidence intervals (CIs) were evaluated using the bootstrap percentiles method (*N* = 1,000). Effects were considered significant if the resulting CI did not contain 0. All continuous variables were grand mean centered to reduce collinearity. As mentioned in the “Procedure” section, any patient missing an ED symptoms assessment at treatment intake or a therapy outcome measure at discharge was not included in the analyses. Due to the software’s fixed distribution requirement, no SWAP-200 data were missing.

## Results

### Relationships Between PDs and Healthy Personality Functioning, Pre-treatment Symptomatic Impairment, and Therapy Outcome

The results showed that Clusters A and B of the SWAP-200 PD scales were positively associated with higher levels of overall ED symptomatic impairment at treatment intake and worse therapy outcome, with Cluster B showing an additional association with more severe baseline bulimic symptoms (see Table 1). Furthermore, SWAP-200 healthy personality functioning was negatively associated with more severe baseline ED and bulimic symptoms, as well as to worse therapy outcome. Contrary to expectations, Cluster C of the SWAP-200 PD scales was not significantly related to pre-treatment ED symptoms or therapy outcome, but showed only a negative association with baseline severity of bulimic symptoms.

**TABLE 1:** Means, standard deviations, and correlations between clusters of SWAP-200 PD scales, healthy personality functioning, baseline eating symptoms, and therapy outcome (*N* = 107).

		**BITE intake^b^**	**EDI-3^c^ intake**	**OQ-45.2^d^ termination**
**SWAP-200^a^ PD scales**	***M* (*SD*)**	**16.07 (6.04)**	**5.19 (0.72)**	**72.22 (27.07)**
Cluster A	46.82 (6.54)	–0.014	0.243*	0.287**
Cluster B	45.47 (4.69)	0.384***	0.210*	0.224*
Cluster C	49.34 (6.46)	−0.387**	–0.057	0.165
Healthy personality functioning	50.87 (6.56)	−0.222*	−0.213*	−0.522***

^*a*^Shedler-Westen Assessment Procedure-200 (SWAP-200; Westen and Shedler, 1999a,b); ^*b*^Bulimic Investigatory Test, Edinburgh (BITE; Orlandi et al., 2005); ^*c*^Eating Disorder Inventory-3 (Garner, 2004); ^*d*^Outcome Questionnaire-45.2 total score (Lambert et al., 2010).

*p ≤ 0.05; **p ≤ 0.01; *** p ≤ 0.001.

### Comparisons Between AN and BN Patients in Pre-treatment ED Symptomatic Impairment and Therapy Outcome

To assess differences between AN and BN patient groups in pre-treatment overall ED and bulimic symptoms and therapy outcome, after controlling for patients’ age and BMI as covariates, a MANCOVA was performed. The results revealed no significant effects for patients’ age [Wilks’ lambda = 0.93; *F*(1, 105) = 1.91; *p* = 0.13, η*_*p*_*^2^ = 0.06] or BMI [Wilks’ lambda = 0.97; *F*(1, 105) = 0.67; *p* = 0.57, η*_*p*_*^2^ = 0.02]. Furthermore, there were no significant differences between AN and BN participants in EDI-3 overall symptomatic impairment at treatment intake [*F*(1, 105) = 2.46; *p* = 0.07, η*_*p*_*^2^ = 0.07)] or OQ-45.2 total score at discharge [*F*(1, 105) = 0.87; *p* = 0.46, η*_*p*_*^2^ = 0.03)]. However, patient groups significantly differed in terms of the baseline BITE severity of bulimic symptoms [*F*(1, 105) = 6.75; *p* < 0.001, η*_*p*_*^2^ = 0.19)]. More specifically, BN patients showed higher average levels of baseline bulimic symptomatology (*M*_*BITE*_ = 19.97, *SD* = 6.38) than AN patients (*M*_*BITE*_ = 13.40, *SD* = 5.52).

### Personality, Pre-treatment ED Symptoms, and Therapy Outcome: A Mediation Analysis

**FIGURE 1:**
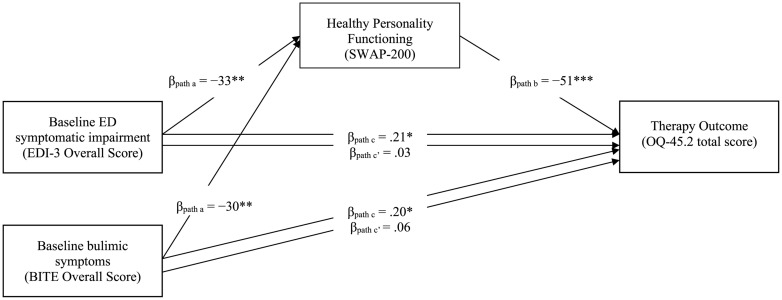
Mediation Model with SWAP-200 Healthy Personality Functioning as a Mediator of the Effect of Bulimic and Overall Eating Symptoms at Intake on Therapy Outcome (*N* = 107). EDI-3 = Eating Disorder Inventory-3 (Garner, 2004); SWAP-200 = Shedler-Westen Assessment Procedure-200 Healthy Personality Functioning Scale (Westen and Shedler, 1999a,b); BITE = Bulimic Investigatory Test, Edinburgh (BITE; Orlandi et al., 2005); OQ-45.2 = Outcome Questionnaire-45.2 total score (Lambert et al., 2010). Confidence intervals computed using bootstrap percentiles.

The first two mediational models included the SWAP-200 healthy personality functioning as a mediator in the relationship between baseline symptomatic impairment and therapy outcome (see Figure 1). The results of the first mediation analysis showed that the total effect (path “*c*”) of the baseline EDI-3 overall score on therapy outcome was significant (β = 0.21, *p* < 0.05). Significant coefficients of path “*a*” (EDI-3 overall score on healthy personality functioning; β = −0.33, *p* = 0.002) and path “*b*” (healthy personality functioning on therapy outcome; β = −0.51, *p* < 0.001) were also found. The point estimate of the indirect effect between pre-treatment EDI-3 and therapy outcome through healthy personality functioning (path “*a^∗^b*”) was.033 (*SE* = 0.01, β = 0.17, *p* = 0.012), and the bootstrapped 95% CIs did not include 0 (0.001 −0.006), indicating that the indirect effect of healthy personality functioning was significant. In addition, the direct effect of baseline EDI-3 score on therapy outcome (path “*c’*”) was not significant after controlling for the mediator (β = 0.03, *p* = 0.73). Figure 1 also shows that, in the second mediation model, healthy personality functioning mediated the relationship between baseline BITE score and therapy outcome (path “*a^∗^b*”) (β = 0.15, *p* = 0.002), as the resulting bootstrapped CIs did not contain 0 (0.006–0.033). The inclusion of SWAP-200 healthy personality functioning as a mediator implied a considerable reduction in the effect of baseline bulimic symptoms on therapy outcome, making it no longer significant (β = 0.06, *p* = 0.48).

**FIGURE 2:**
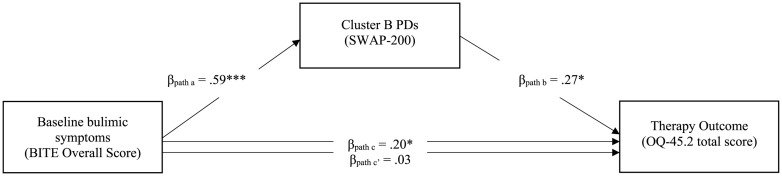
Mediation Model with Cluster B of the SWAP-200 PD Scales as a Mediator of the Effect of Overall Bulimic Symptoms at Intake on Therapy Outcome (*N* = 107). SWAP-200 = Shedler-Westen Assessment Procedure-200 (Westen and Shedler, 1999a,b); BITE = Bulimic Investigatory Test, Edinburgh (BITE; Orlandi et al., 2005); OQ-45.2 = Outcome Questionnaire-45.2 total score (Lambert et al., 2010). Confidence intervals computed using bootstrap percentiles.

Contrary to expectations, our findings shows that the mediated indirect effect (path “*a^∗^b*”) of Cluster A of the SWAP-200 PD scales in the relationship between baseline EDI-3 score and therapy outcome was not significant (β = 0.05, *p* = 0.28). However, in the fourth mediation model, Cluster B of the SWAP-200 PD scales was found to mediate the link between baseline BITE score and therapy outcome (see Figure 2). The findings indicated that the total effect (path “*c*”) was significant (β = 0.20, *p* = 0.044), involving significant coefficients for both path “*a*” (β = 0.59, *p* < 0.001) and path “*b*” (β = 0.27, *p* = 0.036). The point estimate of the indirect effect between pre-treatment BITE and therapy outcome through the Cluster B score (path “*a^∗^b*”) was.022 (*SE* = 0.01, *p* = 0.038), and the bootstrapped 95% CI did not include 0 (0.001–0.044), indicating that the mediated indirect effect was significant. The direct effect of baseline BITE score on therapy outcome (path “*c’*”) was no longer significant (β = 0.03, *p* = 0.75). Finally, Cluster C of the SWAP-200 PD scales did not show any mediator effect (path “*a^∗^b*”) in the relationship between pre-treatment EDI-3 overall score or BITE total score and therapy outcome (β = 0.02, *p* = 0.37 and β = −0.006, *p* = 0.79, respectively).

## Discussion

The main aim of the present study was to investigate whether overall healthy personality functioning and features of clusters of PDs, assessed dimensionally by the SWAP-200, could mediate the relationship between baseline ED and bulimic symptomatic impairment and therapy outcome at discharge from an ED residential treatment program. Despite preliminary findings of the mediator role of personality with respect to other patient variables in ED samples (Eggert et al., 2007; Münch et al., 2016) and other clinical populations (e.g., Verona et al., 2005; Okubo et al., 2017), the indirect effects of PDs on treatment response in ED patients remains under-researched.

The present study found that: (a) features of PDs in Clusters A and B, but not Cluster C, related to more severe baseline symptomatic presentation and worse therapy outcome, whereas healthy personality functioning showed the inverse associations; (b) the DSM-5 derived categorical ED diagnoses of AN and BN did not significantly differ in terms of ED symptomatic impairment at treatment intake and therapy outcome at discharge, except with respect to higher levels of binge eating and purging behaviors in BN patients; and (c) healthy personality functioning mediated the relationship between baseline symptom severity and therapy outcome, and the association between baseline bulimic symptoms and treatment outcome; furthermore, Cluster B PDs mediated the link between baseline binge eating and purging behaviors and therapy outcome, whereas PDs in Clusters A and C did not show any significant mediating effects.

The identification of significant mediators in ED treatment may help us to identify which intervention will work best for each patient, and under what conditions (Roth and Fonagy, 2005). Findings of a mediator effect of Cluster B symptoms on therapy outcome, over and above the direct effect of the severity of bulimic symptoms, suggest that interpersonal difficulties, unstable self-image and self-esteem, marked impulsivity, dysfunctional defense mechanisms, and emotion dysregulation might play a relevant role in determining response to ED treatment (Martinussen et al., 2017; Voderholzer et al., 2021). In line with previous empirical evidence, co-occurring Cluster B symptoms in an ED patient may mark greater severity, and thus potentially predict a poorer outcome and less symptomatic change (Steiger et al., 1993; Hessler et al., 2019). Cluster B personality characteristics have also been related to other therapeutic variables, such as a history of psychiatric hospitalization for an ED or other condition (Westen and Harnden-Fischer, 2001) and a greater risk of discharge against medical advice and re-admission following intensive ED treatment (Wildes et al., 2011). These observations seem particularly relevant to intensive treatment settings, such as residential programs, in which these features may lead to a tendency to break rules and thereby limit patients’ adjustment to the structured treatment protocol (Friedman et al., 2016; Muzi et al., 2019, 2020).

On the other hand, the SWAP-200 healthy personality functioning score, which measures psychological strengths such as mature defense mechanisms (e.g., humor), empathic abilities, responsiveness, capacities for relationship and intimacy, nurturance, affective regulation, insight, and reflective capacities (Westen and Shedler, 1999a,b), was found to significantly interact with and reduce the effect of baseline symptomatic impairment in determining therapy outcome. Despite the need to replicate these findings on larger ED samples, it might be possible to hypothesize that targeting these protective factors related to psychological resources and well-being may increase the effectiveness of prevention and intervention programs for this clinical population (e.g., Tomba et al., 2014, 2017). However, contrary to expectations, the present findings did not show any significant mediating effect of PDs in Clusters A and C. This is surprising, due to the literature linking obsessive-compulsive personality traits and perfectionism with negative outcomes in ED patients, particularly those with mainly anorectic/restricting symptoms (Lilenfeld et al., 2006; Crane et al., 2007; Farstad et al., 2016). A possible explanation for this is that the SWAP-200 obsessive-compulsive personality and overall Cluster C PDs slightly differ from the corresponding DSM descriptions, showing some psychological strengths and the highest correlations with the Global Assessment of Functioning Scale (see Westen and Shedler, 1999b). Another potential reason is that the present study explored the mediating role of PD features only at a cluster level, due to the well-known high rates of PD comorbidity (e.g., Lenzenweger et al., 2007; Zimmerman, 2012), which may have masked some relevant findings at the individual PD level. Future research with larger sample sizes should explore the mediating effects of each PD score, especially when measured dimensionally, in line with the growing consensus on the limitations of contemporary categorical conceptualizations of personality (e.g., Widiger, 2007; Westen et al., 2012).

In this vein, another interesting result is that DSM-5 categorical diagnostic categories for EDs did not show adequate discriminant validity in terms of baseline variables, ED-specific symptoms (with the exception of binge eating and purging behaviors), and therapy outcome, in line with the literature (Westen and Harnden-Fischer, 2001; Raykos et al., 2018). Other studies have found that DSM-5 subtypes and their severity specifiers are not reliable indicators of the concurrent severity of ED symptomatic impairment (Smith et al., 2017). Taken together, these findings corroborate the hypothesis that more dimensional, individualized, and personality-based outcome studies may helpfully supplement the categorical framework in which EDs have been traditionally conceptualized. As outlined by Martinez and Craighead (2015), rather than classifying individuals based on the presence or absence of disordered eating behaviors, employing these alternative approaches might lead to improved knowledge of more generalized dysfunctions in psychological processes across several areas of individual functioning, which tend to be stable over time and across situations. These individual characteristics might include impaired mentalizing capacities (e.g., Rothschild-Yakar et al., 2010); difficulties in emotion regulation (Harrison et al., 2010); diminished interpersonal abilities (McAdams et al., 2015); and impaired self-directedness, self-awareness, and self-understanding (Marco et al., 2019). This strategy could be extremely useful for the development of targeted and patient-tailored treatment options to maximize successful outcomes (Norcross and Lambert, 2018).

Several shortcomings and methodological issues of the present study should be noted. First, the data derived from a single multimodal and residential ED program with a predominantly White population of cisgender women, which may limit the generalizability of the findings to other ED treatment settings or populations, especially those with baseline EDs other than AN or BN (e.g., binge eating disorder). Future studies should explore the mediating effects of personality functioning and disorders including a more heterogeneous ED patient sample and less intensive treatment settings (e.g., outpatient and day treatment programs). Second, although the present study adopted a multi-informant perspective, no data are available on the interrater reliability of either the DSM-5 diagnoses or the SWAP-200 evaluations. Future investigations should employ at least two independent raters for the personality assessments, to acquire more reliable data for analysis. With respect to the latter point, future studies should evaluate more comprehensive and reliable outcome indices. Such investigations might explore changes in personality functioning, as well as the potential indirect effects of the therapeutic alliance or therapist effects on ED treatment outcome (e.g., Colli et al., 2016; Lingiardi et al., 2018; Tanzilli et al., 2018; Groth et al., 2020). Lastly, some authors (e.g., Lilenfeld et al., 2006) have noted that it is difficult to differentiate the pathoplasty model from the so-called *predispositional model*. It is likely that if a particular personality trait has served as a predispositional risk factor for an ED, it will continue to operate as a pathoplastic factor over time. The present findings cannot establish any causal relationship between personality, ED symptoms, and therapy outcome; however, it will be important to further explore, via accurate longitudinal data, whether some features of PDs have proper causal significance in severe eating pathologies.

Despite these limitations, the present findings suggest that personality functioning and disorders may predict baseline symptomatic expression and treatment outcome in EDs, and that a deeper understanding of patient-related moderators and mediators of outcome should be enhanced to improve treatment effectiveness (Linardon et al., 2017). Most ED treatment guidelines share the view that patients’ individual differences, with respect to symptom severity, treatment history, and comorbid psychopathology, should be clearly acknowledged to guide the selection of adequate psychosocial interventions within a stepped-care therapeutic approach (NICE, 2004; American Psychiatric Association, 2006). Thus, future research is needed to clarify the optimal integration of personality variables into ED treatment. Only then will we be able to say if shifting from a “one-size-fits-all” to a “person(ality)-centered” approach may represent a relevant advancement over the *status quo*.

## Data Availability Statement

The raw data supporting the conclusions of this article will be made available by the first author, without undue reservation.

## Ethics Statement

The studies involving human participants were reviewed and approved by the Research Ethics Committee of the Department of Dynamic and Clinical Psychology, Sapienza University of Rome, Italy (Reference number: 0000398). The patients/participants provided their written informed consent to participate in this study.

## Author Contributions

LM and VL conceived the study hypotheses, drafted the first version of the present manuscript, and contributed to the data analysis. LT, AF, and MR contributed to data collection and assessment and extensively revised the manuscript. All authors critically revised the manuscript and approved the final version to be published.

## Conflict of Interest

The authors declare that the research was conducted in the absence of any commercial or financial relationships that could be construed as a potential conflict of interest.
